# The Impact of Comorbidities on Pulmonary Function Measured by Spirometry in Patients After Percutaneous Cryoballoon Pulmonary Vein Isolation Due to Atrial Fibrillation

**DOI:** 10.3390/jcm14155431

**Published:** 2025-08-01

**Authors:** Monika Różycka-Kosmalska, Marcin Kosmalski, Michał Panek, Alicja Majos, Izabela Szymczak-Pajor, Agnieszka Śliwińska, Jacek Kasznicki, Jerzy Krzysztof Wranicz, Krzysztof Kaczmarek

**Affiliations:** 1 Department of Electrocardiology, Medical University of Lodz, 92-213 Lodz, Poland; monika.rozycka-kosmalska@umed.lodz.pl (M.R.-K.); jerzy.wranicz@umed.lodz.pl (J.K.W.); krzysztof.kaczmarek@umed.lodz.pl (K.K.); 2 Department of Clinical Pharmacology, Medical University of Lodz, 90-153 Lodz, Poland; 3 Department of Internal Medicine, Asthma and Allergy, Medical University of Lodz, 90-153 Lodz, Poland; michal.panek@umed.lodz.pl (M.P.); alicja.majos@umed.lodz.pl (A.M.); 4 Department of General and Transplant Surgery, Medical University of Lodz, 90-153 Lodz, Poland; 5 Department of Nucleic Acid Biochemistry, Medical University of Lodz, 92-213 Lodz, Poland; izabela.szymczak@umed.lodz.pl (I.S.-P.); agnieszka.sliwinska@umed.lodz.pl (A.Ś.); 6 Department of Internal Medicine, Diabetology and Clinical Pharmacology, Medical University of Lodz, 92-213 Lodz, Poland; jacek.kasznicki@umed.lodz.pl

**Keywords:** atrial fibrillation, pulmonary vein isolation, cryoballoon ablation, spirometry, lung function, comorbidities

## Abstract

**Background/Objectives:** Pulmonary vein isolation (PVI) via cryoballoon ablation (CBA) is a recommended therapeutic strategy for patients with symptomatic paroxysmal and persistent atrial fibrillation (AF) who are refractory to antiarrhythmic drugs. Although PVI has demonstrated efficacy in reducing AF recurrence and improving patients’ quality of life, its impact on respiratory function is not well understood, particularly in patients with comorbid conditions. The aim of the study was to search for functional predictors of the respiratory system in the process of evaluating the efficiency of clinical assessment of CBA in patients with AF. **Methods:** We conducted a prospective study on 42 patients with symptomatic AF who underwent CBA, assessing their respiratory function through spirometry before and 30 days after the procedure. Exclusion criteria included pre-existing lung disease and cardiac insufficiency. The impact of variables such as body mass index (BMI), coronary artery disease (CAD) and heart failure (HF) on spirometry parameters was analyzed using statistical tests. **Results:** No significant changes were observed in overall post-PVI spirometry parameters for the full cohort. However, post hoc analyses revealed a significant decline in ΔMEF_75_ in patients with CAD and BMI ≥ 30 kg/m^2^, whereas ΔFEV_1_/FVCex was significantly increased in patients with HF, as well as in patients with ejection fraction (EF) < 50%. **Conclusions:** CBA for AF does not universally affect respiratory function in the short term, but specific subgroups, including patients with CAD and a higher BMI, may require post-procedure respiratory monitoring. In addition, PVI may improve lung function in patients with HF and reduced EF.

## 1. Introduction

Atrial fibrillation (AF) is the most common cardiac arrhythmia, characterized by rapid, irregular atrial excitation that leads to dyssynchronous atrial contraction and uneven ventricular activation [1]. It affects over 59 million people worldwide, with prevalence projected to rise significantly by 2050 [2]. AF increases the risk of stroke, heart failure, and death and impairs quality of life [3]. AF is classified as duration into paroxysmal (PAF), persistent (AF-PE), and permanent with treatment goals focusing on symptom control, reducing long-term complications, and improving quality of life. Catheter ablation (CA) prevents AF recurrences, reduces AF burden, and improves the quality of life in symptomatic PAF and AF-PE patients who do not tolerate or do not respond to antiarrhythmic drugs [4]. One of the methods of CA is pulmonary vein isolation (PVI) using a balloon-mounted cryoablation system. Complete pulmonary vein occlusion during balloon ablation has been demonstrated to predict successful electrical isolation [5]. The European Society of Cardiology and Heart Rhythm Society Guideline identify PVI as the main strategy for treating symptomatic, drug-refractory AF. They also note that cryoballoon CA (CBA) can be an effective alternative to the traditional radiofrequency ablation. PVI is usually performed via the inferior vena cava through the access of the femoral veins [6,7].

The modern transvenous cryocatheter system includes a deflectable hollow shaft with a cooling tip, ring electrodes, and a thermocouple. Ablation is performed by delivering pressurized cryorefrigerant from an external console through a thin injection tube. The refrigerant absorbs heat from the heart tissue via convective cooling, causing cellular injury through ice crystal formation and microcirculatory failure [8,9].

PVI with high-energy ablation is effective for AF, with success rates of 50–95%. However, it is invasive and carries risks such as cardiac tamponade, thromboembolism, pulmonary vein stenosis, atrioesophageal fistula, and other complications [10,11,12]. Mugnai et al. reported a serious adverse event rate of 2.9%, including vascular complications (1.1%), tamponade (1.0%), and thromboembolism (0.3%). Rare complications like atrioesophageal fistula and nerve palsy occurred in 0.1% of cases, with no procedure-related deaths. The overall complication rate was similar for both radiofrequency and cryoballoon techniques [13]. A multicenter study in Poland found a low adverse event rate of 6.4%, including mainly local complications, and a low incidence of post-procedure atrial flutter or tachycardia (5%). Most procedures used radiofrequency ablation (89%) [14]. There is a lack of data on the effect of CBA on lung function. Furthermore, it is not known whether or to what extent this relationship is influenced by comorbidities and cardiac dysfunction.

The aim of this study was to search for functional predictors of the respiratory system in the process of evaluating the efficiency of clinical assessment of CBA in patients with AF.

## 2. Materials and Methods

### 2.1. Study Population

This study included a total of 42 consecutive patients (29 males and 13 females) aged between 36 and 77 years (mean: 51.25; range: 36–77) who were qualified for PVI as the first interventional antiarrhythmic treatment for symptomatic atrial fibrillation at the Department of Electrocardiology, Central University Hospital of the Medical University of Lodz, between 2020 and 2021. The inclusion criteria covered the following: (1) diagnosed FA (PAF/AF-PE) before PVI; (2) age over 18 years; (3) indications for the PVI procedure (lack of effectiveness of antiarrhythmic therapy, side effects caused by antiarrhythmic therapy, a serious threat to the patient’s life posed by arrhythmia); (4) presence of an active inflammatory condition; (5) cryoballoon as the primary planned PVI technique; (6) eligibility and willingness for spirometry testing; (7) signed informed consent to participate in this study. The exclusion criteria included the following: (1) lung diseases such as pneumonia, bronchial asthma, chronic obstructive pulmonary disease (COPD), and lung cancer; (2) phrenic nerve injury during CB-PVI; (3) previous PVI; (4) clinical signs and symptoms of respiratory insufficiency. PAF was defined according contemporaneous guidelines [4]. All patients provided written informed consent. This study was approved by the Medical University of Lodz Committee on the Ethics of Research in Human Experimentation (approval number RNN/182/17/KE) and was conducted in accordance with the Helsinki Declaration for human research. This study was not registered as a clinical trial because it was a non-interventional study that only added a respiratory function test to routine medical examinations. Spirometry was performed in each qualified patient before PVI and repeated 30 days after the procedure.

### 2.2. Spirometry

Respiratory function tests were performed according to European Respiratory Society (ERS) and the American Thoracic Society (ATS) standards [15]. All spirometry measurements were performed using the Lungtest 1000 device (producent: MES Sp. z o.o. of Krakow, Poland). Prior to testing, patients were instructed to breathe normally, and each measurement was repeated at least three times to ensure reproducibility. The best score of three spirometry readings was selected for analysis, following the guidelines of the Polish Society of Lung Diseases [16]. During spirometry, the following parameters were assessed: FEV_1_/FVC_ex_ (forced expiratory volume during the first second of expiration to forced vital capacity), FEV_1_ (forced expiratory volume during the first second of expiration), FVC_ex_ (expiratory forced vital capacity), PEF (peak expiratory flow), and MEF75, MEF50, and MEF25 (maximal expiratory flow at 75%, 50%, and 25% of FVC) to assess small and large airway function. For each parameter, the percentage of predicted values was calculated based on reference values adjusted for age, sex, height, and ethnicity, according to standard reference equations. The predicted values served for comparison to assess the degree of airway obstruction or restriction in individual patients.

### 2.3. Pulmonary Vein Isolation

PVI was performed percutaneously using the cryoballoon technique [17], according to the standard of care at our institution. The procedure began with the insertion of two vascular sheaths into the right femoral vein under sterile conditions. Prior to the transseptal puncture procedure, transesophageal echocardiography (TEE) was performed to exclude the presence of a thrombin in the left atrial appendage, thereby reducing the risk of embolic complications. The transseptal puncture was then carried out under combined guidance with fluoroscopy and TEE to ensure precise access to the left atrium. A dedicated transseptal sheath for the cryoballoon procedure was inserted into the left atrium and continuously flushed with heparinized saline to prevent clot formation. Following standard preparation, the balloon catheter was advanced into the pulmonary veins. The catheter was positioned to occlude each pulmonary vein individually, confirmed by contrast injection and imaging. Once proper occlusion was achieved, cryoablation was performed for a duration ranging from 180 to 300 s, with the application time adjusted based on the minimum temperature reached during each freeze cycle—with colder temperatures typically requiring shorter application times to optimize safety and efficacy. Throughout the entire procedure—from the transseptal puncture to the removal of catheters from the left atrium—the activated clotting time (ACT) was maintained above 300 s to minimize thromboembolic risk. The ACT was monitored regularly and adjusted with heparin infusion as needed.

### 2.4. Statistical Analysis

The measured respiratory parameters were expressed as medians (quartile 1; quartile 3). The null hypothesis that the data in the tested population would be normally distributed was rejected using the Shapiro–Wilk test. The relationship between respiratory parameters measured before and 30 days after ablation was evaluated using Wilcoxon signed-rank two-tailed test. The differences in the spirometry parameters, defined as the “Δ = (value on day 30)—(value on day 0)” for each spirometry parameter, analyzed in relation to selected coexisting factors, were assessed using the Mann–Whitney U test or Kruskal–Wallis test in the case of multiple comparisons. All analyses were carried out using the Statistica 13PL software (StatSoft Polska Sp. z o.o. Cracow, Poland). The significance level was assumed to be 0.05 in all the statistical tests.

## 3. Results

Out of a total of 42 consecutive patients included in this study, only 27 patients completed the entire study, of whom 14 patients did not return for follow-up visits and 1 patient died during the study without any relation to the PVI procedure. Median age in the whole group was 58 years (IQR, 48.0–69.0; min.-max., 38.0–77.0) with a median body mass index (BMI) of 28.7 kg/m^2^ (IQR 25.7–32.5, min.-max. 21.5–48.4) and median EF of 56.0% (IQR, 52.0–62.0%; min.-max., 22.0–69.0%).

The characteristics of patients who completed the entire study are presented in Table 1.

The comparison of spirometry parameters pre-PVI and 30 days after PVI for the whole study group is presented in Table 2; no statistically significant differences were observed in this approach to this study.

**Figure 1:**
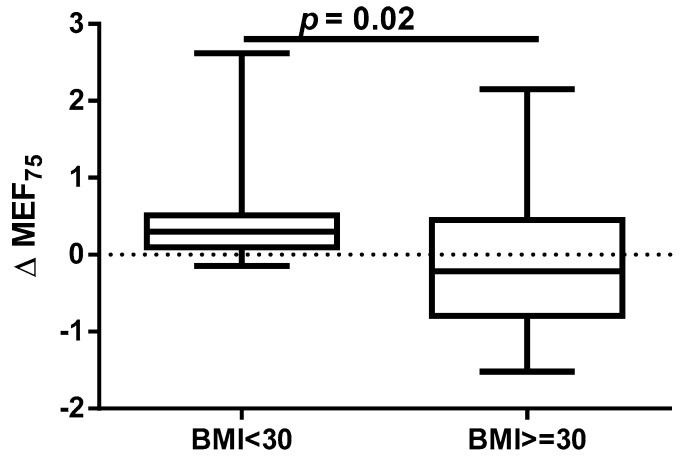
Δ% MEF_75_ in subgroups according to BMI class.

**Figure 2:**
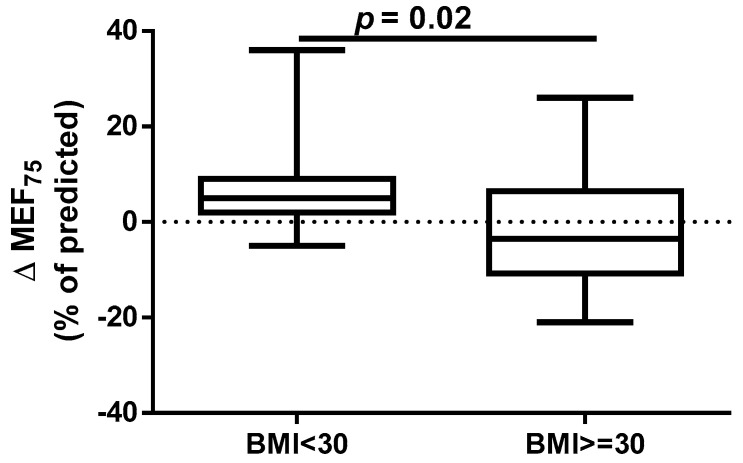
Δ% MEF_75_ (% of predicted) in subgroups according to BMI class.

**Figure 3:**
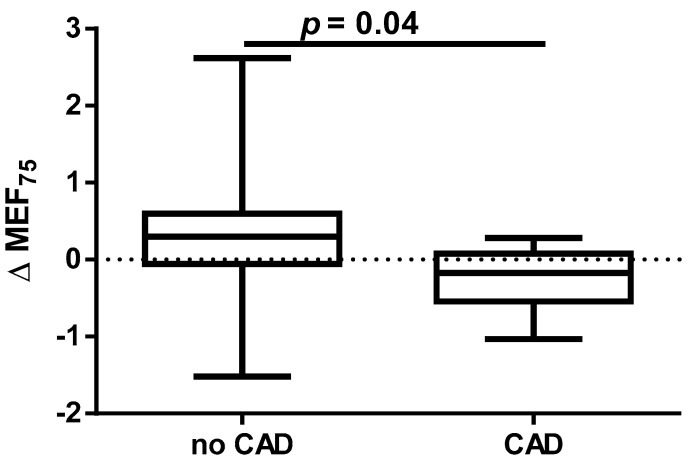
Δ% MEF_75_ in subgroups according to CAD presence.

**Figure 4:**
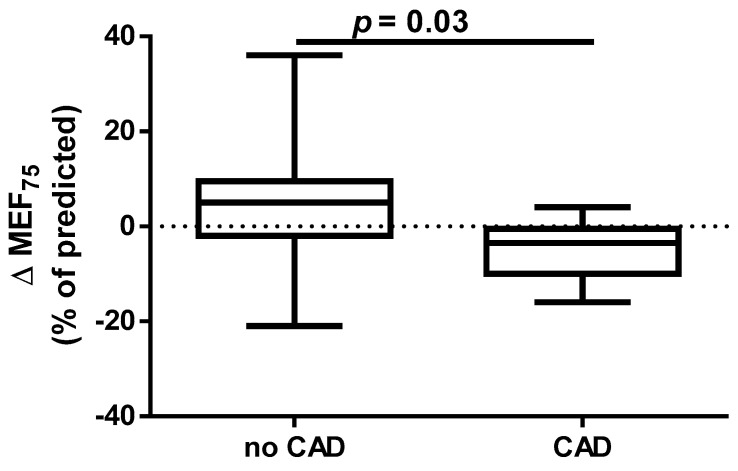
Δ% MEF_75_ (% of predicted) in subgroups according to CAD presence.

**Figure 5:**
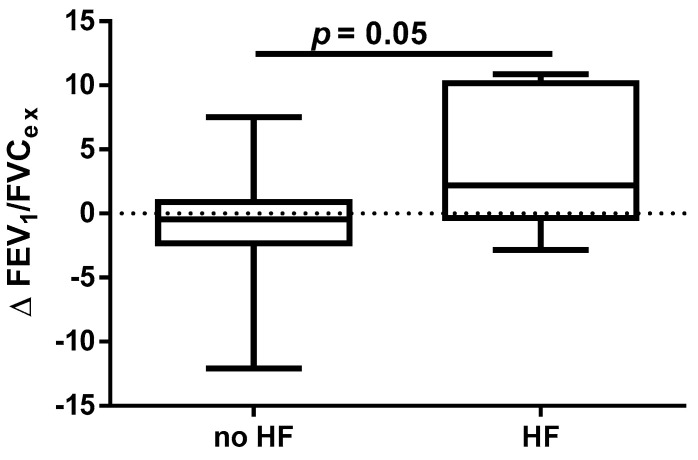
Δ% FEV_1_/FVC_ex_ in subgroups according to HF presence.

**Figure 6:**
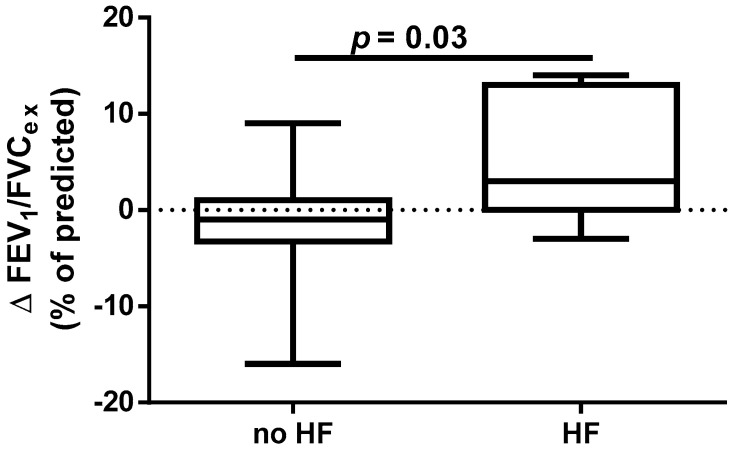
Δ% FEV_1_/FVC_ex_ (% of predicted) in subgroups according to HF presence.

**Figure 7:**
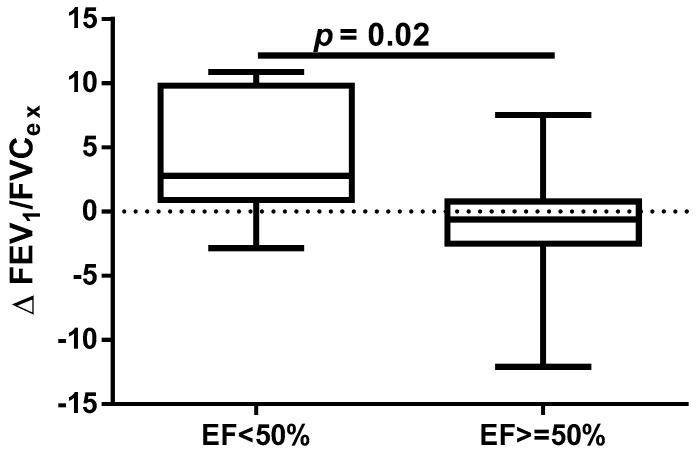
Δ% FEV_1_/FVC_ex_ in subgroups according to EF value.

**Figure 8:**
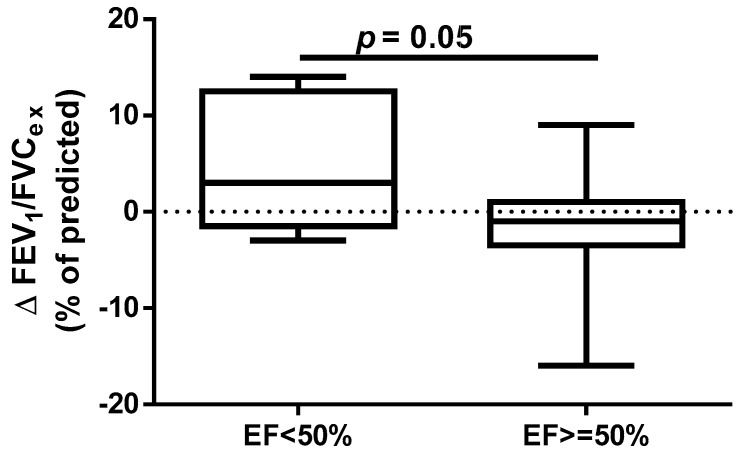
Δ% FEV_1_/FVC_ex_ (% of predicted) in subgroups according to EF value.

A different approach (Δ of each spirometry parameter) was applied for analysis according to the occurrence of comorbidities. The analysis of Δ of the assessed spirometry parameters before and 30 days after PVI in relation to BMI, coronary artery disease (CAD), heart failure (HF), and ejection fraction (EF) value was performed (data presented in Supplementary Materials Tables S1–S4). As shown in Figure 1 and Figure 2, among the tested parameters, Δ MEF75 (l/s) (*p* = 0.02) was significantly elevated in the group with BMI < 30 kg/m^2^ as compared to the group with BMI ≥ 30 kg/m^2^. A similar statistically significant difference was found in Δ MEF75 (% of predicted) (*p* = 0.02). As shown in Figure 3 and Figure 4, in the group with CAD, Δ MEF75 (l/s) (*p* = 0.04) and Δ MEF75 (% of predicted) (*p* = 0.03) were markedly lower compared to those in the group without CAD. Regarding the comparison of the group with HF versus the group without HF, presented in Figure 5 and Figure 6, Δ FEV_1_/FVCex (*p* = 0.05) and Δ FEV_1_/FVCex (% of predicted) (*p* = 0.03) showed a significant increase in the group with HF. Figure 7 compares the parameters between the group with EF < 50% and the group with EF ≥ 50%. The results reveal a significant increase in Δ FEV_1_/FVCex (*p* = 0.02) in the group with EF < 50%, suggesting that the change in this parameter was notably greater in patients with reduced ejection fraction. Similarly, Figure 8 depicts the comparison of parameters between the two groups. The findings demonstrate a significant increase in Δ FEV_1_/FVCex (% of predicted) (*p* = 0.05) in the EF < 50% group, indicating that the change in this parameter was also significantly higher in patients with lower EF.

We did not observe statistically significant differences for other comorbidities such as type 2 diabetes mellitus (T2DM), dyslipidemia (DLP), and hypertension (HA) as well as EHRA (European Hearth Rhythm Association) score and AF type (Tables S5–S9 in Supplementary Materials).

## 4. Discussion

Studies have shown a correlation between impaired lung ventilation, as confirmed by spirometry, and the risk of AF. This applies to individuals both with and without diagnosed lung diseases like COPD or bronchial asthma. Furthermore, it has been shown that this relationship is independent of other cardiovascular risk factors and concerns reduced values of FEV_1_, FVC, and the FEV_1_/FVC ratio, as well as the percentage of predicted FEV_1_ and FVC [18,19,20,21,22,23]. The exact mechanisms linking decreased lung function and AF are unclear, but potential factors include hypoxia, systemic inflammation, increased sympathetic activity, and treatments like beta-2 agonists or steroids [24]. Recent research suggests that ectopic beats triggering AF often originate in the pulmonary veins, possibly due to changes in gas composition or pulmonary hypertension. Even mild to moderate reductions in lung function have been associated with AF, and reduced lung capacity is also an independent predictor of coronary artery disease and stroke. The connection may involve shared pathways like atherosclerosis, influenced by early-life exposures or vascular and airway diseases. The close anatomical and physiological relationship between the lungs, the heart, and blood vessels suggests that damage to one component can affect overall cardiovascular health [25,26,27,28,29,30].

However, there is no data assessing the effect of CA on lung function in patients with AF. In a study conducted by Oguri et al., it was found that CA performed by various techniques, including radiofrequency catheter ablation (RFCA), CBA, hot balloon ablation, and laser balloon-mediated ablation, significantly improved VC, FVC, and PEF in patients with PAF and non-PAF defined as persistent AF (defined as AF-PE or long-standing AF-PE during 6-month observation period). In the patients with PAF, a significant increase in FEV_1_ was observed. CBA significantly improved VC and FVC. A significant improvement in these pulmonary parameters was specifically observed in patients with PAF treated with a CBA protocol but not with RFCA or hot balloon ablation. A significant decrease in FEV_1_ was observed with hot balloon ablation. Significant improvements in pulmonary parameters were seen specifically in patients with PAF who underwent CBA [31]. Additionally, we conducted an analysis of the impact of the CBA procedure on spirometry parameters to determine whether it might cause ventilation disorders in short-term observation and, if present, the possibility of applying appropriate pharmacotherapy. However, it should be emphasized that we did not find a significant effect of selected ablation techniques on the spirometry results in the entire cohort of patients with PAF and AF-PE. It is worth noting that, to minimize confounding factors, we excluded patients with previously diagnosed lung diseases and smokers from our study.

It has been proven that, apart from lung diseases such as asthma or COPD, factors such as age, sex, race, HA, congestive HF, CAD, obesity, T2DM, DLP, physical inactivity, obstructive sleep apnea, and smoking are associated with increased odds of developing AF [32,33,34,35,36,37]. Moreover, the Prospective Global GLORIA-AF Registry revealed that these comorbidities influence the management and long-term prognosis of patients with AF [38]. Despite the established association between cardiovascular risk factors such as obesity, dyslipidemia, T2DM, HA, HF, CAD, and the presence of AF, data assessing the impact of these diseases on the efficacy and safety of CA, particularly PVI, in both PAF and AF-PE groups are still lacking.

An annual observation conducted by Sargent et al. showed that the PVI procedure, performed using either CBA or radiofrequency ablation, was more successful in patients with lower weight and BMI, particularly those with a BMI < 27 kg/m^2^ and in those without obstructive sleep apnea. The only independent predictor of the primary outcome (successful isolation of the pulmonary veins and freedom from AF without repeated ablation or ongoing antiarrhythmic therapy at 12 months) was BMI. Furthermore, over 75% of complications occurred in patients with a BMI ≥ 27 kg/m^2^ [39]. It should be emphasized that, despite the proven association between obesity and long-term success of pulmonary vein isolation performed using transcatheter radiofrequency for drug-refractory AF [40], it has been shown that for obese patients, CBA is as safe and effective as RFCA. The significantly shorter procedure time for CBA may minimize potential obesity-related complications. However, the lower contrast medium quantity and fluoroscopy dose in RFCA should be considered. AF recurrence rates are comparable between CBA and RFCA [41]. However, some data indicate that CBA in overweight and obese patients is safe, with similar levels of complications and AF recurrence rates compared to those in patients of healthy weight. However, obese patients and operators are exposed to higher radiation doses [42,43,44]. Our results indicate that a BMI ≥ 30 kg/m^2^ significantly impairs respiratory ventilation in patients undergoing the CBA procedure, due to significant decrease in the MEF_75_ and % of predicted MEF_75_ values. It is important to note that the MEF_75_ value reflects the functional state of the medium and small airways, and a decrease in this indicator is common even in the early stages of cardiovascular diseases [45].

Similar observations were also made for patients with T2DM who underwent CBA [46]. It has been demonstrated that T2DM is an independent predictor of AF recurrence after catheter ablation, particularly in patients with persistent AF [47]. Recent data indicate that baseline HbA1c level is an independent predictor of AF recurrence following cryoablation, both in patients with and without diabetes [48]. Moreover, both existing T2DM and newly diagnosed T2DM were identified as independent risk factors for AF recurrence following ablation. This study underscores the urgent need for careful management of diabetes mellitus in individuals undergoing AF ablation to reduce the likelihood of arrhythmic relapse [49]. Considering the significant number of patients with T2DM and obesity, it is important to note the possibility of undiagnosed obstructive sleep apnea (OSA) in the analyzed group [50,51]. It is worth emphasizing that OSA may significantly impact spirometry parameters, and due to factors such as regions of interest (ROIs) and hypoxemia, it could lead to AF [52,53]. Nevertheless, it is worth emphasizing that our study did not demonstrate any influence of T2DM on spirometry parameters.

Despite the proven beneficial effect of CBA for AF in patients with heart failure with mid-range ejection fraction (HFmrEF) and heart failure with preserved ejection fraction (HFpEF) [54,55], no studies have assessed the influence of heart function on short- and long-term prognosis after this procedure. In our study, we found a beneficial effect of the CBA procedure on spirometry parameters such as FEV_1_/FVC_ex_ value in the group of patients with HF and % of predicted FEV_1_/FVC_ex_. A similar positive effect was noted in the group of patients with EF <50%, where a significant improvement in the index was observed. It is highlighted that this observation is clinically significant because, in patients with HF, pulmonary function assessment, particularly spirometry, can serve as a non-invasive and sensitive method for detecting and monitoring early pulmonary congestion. This approach could complement the measurement of natriuretic peptides (BNP and NT-proBNP). Such diagnostic procedures provide an opportunity for early pharmacological and non-pharmacological interventions. The respiratory system is an integral part of the cardiorespiratory interactions in HF, which is why pulmonary function tests, including spirometry and other methods (e.g., diffusing capacity of the lungs for carbon monoxide—DLCO measurement), should be considered complementary to clinical assessment, echocardiography, cardiac biomarkers such as natriuretic peptides, and chest imaging. Furthermore, pulmonary function parameters provide prognostic information in patients with HF and can assist in treatment decisions. Therefore, spirometry and DLCO should be recommended as part of the initial diagnosis and monitoring of patients with HF. Longer follow-up periods, such as one year, using pulmonary function tests may help identify risk markers for the CBA procedure [56].

Data on the impact of CAD on the effectiveness and safety of CBA suggest that CBA is effective for treating AF in patients with stable CAD. The presence of CAD does not appear to affect AF recurrence after CA [57]. In our study, we demonstrated for the first time that the presence of CAD before PVI is a risk factor for deterioration of lung ventilation during a 30-day follow-up after PVI according to a significant reduction in MEF_75_ and the % of the predicted FEV_1_/FVC_ex_ value. We did not find any significant effect of HA and DLP, as well as the type of AF and EHRA score on spirometry parameters within 30 days after PVI.

### Study Limitations

A significant limitation of this study is the small sample size, which is largely due to the limited access to various PVI techniques and the coexistence of lung diseases in patients undergoing ablation procedures. Another limitation is the relatively short observation period, but this is related to the focus on assessing the indications for the rapid diagnosis of ventilation disorders in patients after PVI, as well as the ability to implement appropriate pharmacotherapy as soon as possible. Therefore, we plan a long-term (one-year) follow-up of patients participating in this study to assess PVI complications associated with possible pulmonary remodeling, taking into account comorbidities and cardiometabolic risk markers.

It should also be mentioned that this study excluded individuals with known lung disease and smokers, which may be appropriate for internal validity but reduces the generalizability to the typical AF population. Additionally, the presence of OBS and its correlation with T2DM and obesity represent a major unmeasured variable that could substantially bias the respiratory results. Moreover, time to ablation is a crucial factor in assessing the consequences of PVI. Available data suggest that a shorter time to ablation (≤1 year) is associated not only with a lower risk of repeat ablation and new cardioversion but also with a reduced risk of cardiovascular hospitalization compared to late ablation (>1 year). Additionally, shorter time to ablation correlates with a better CHA_2_DS_2_-VASc score, lower incidence of HF, and higher ejection fraction (EF). Given the observed associations with PVI, long-term follow-up appears warranted [58].

## 5. Conclusions

In our study, we observed that CBA for AF was not associated with significant changes in spirometry parameters during a one-month observation period. However, we observed that in obese patients, as well as in those with CAD, there were indications that post-procedural spirometry parameters might deteriorate significantly. This preliminary observation suggests the potential need for close monitoring of respiratory function in these patient groups following the procedure to enable timely implementation of appropriate pharmacotherapy. On the other hand, there is a hypothesis that PVI may have the potential to improve lung function in patients with HF and reduced EF. Given the limited sample size and the preliminary nature of these findings, further investigation in larger cohorts with extended follow-up is necessary to better understand these associations and evaluate possible preventive and therapeutic strategies for at-risk populations.

## Figures and Tables

**Table 1:** Patient sex and clinical characteristics.

Parameter	N (%)
Sex	
Female	9 (33.3%)
Male	18 (66.7%)
Comorbidities	
HA	21 (77.7%)
HF	5 (18.5%)
CAD	6 (22.2%)
T2DM	11 (40.7%)
BMI ≥ 30 kg/m^2^	12 (44.4%)
DLP	18 (66.6%)
EF ≥ 50%	21 (77.7%)
AF type (PAF/AF-PE)	18/9 (66.6/33.3%)
EHRA score	
I–II	11 (40.7%)
III–IV	16 (59.2%)

AF-PE—persistent atrial fibrillation; BMI—body mass index; CAD—coronary artery disease; DLP—dyslipidemia; EF—ejection fraction; EHRA—European Hearth Rhythm Association; HA—hypertension arterialis; HF—heart failure; PAF—paroxysmal atrial fibrillation; T2DM—type 2 diabetes mellitus.

**Table 2:** Spirometry parameter values before and 30 days after PVI.

Spirometry Parameter	Before PVI	30 Days After PVI	*p* *
	Median (Q1: Q3)	Median (Q1: Q3)	
FEV_1_/FVC_ex_ (%)	75.78 (69.93; 81.34)	76.68 (70.71; 80.45)	0.94
FEV_1_ (l)	3.00 (2.22; 3.55)	2.97 (2.37; 3.37)	0.18
FVC_ex_ (l)	4.02 (2.99; 4.66)	3.88 (3.04; 4.62)	0.23
PEF (l/s)	5.61 (4.70; 6.86)	5.56 (4.99; 6.40)	0.89
MEF_75_ (l/s)	5.04 (4.21; 6.27)	5.24 (3.93; 5.92)	0.15
MEF_50_ (l/s)	3.10 (2.26; 4.37)	2.98 (2.30; 4.11)	0.17
MEF_25_ (l/s)	0.93 (0.73; 1.31)	0.98 (0.68; 1.18)	0.13
FEV_1_/FVC_ex_ (% of predicted)	96.00 (89.00; 103.00)	97.00 (92.00; 104.00)	0.64
FEV_1_ (% of predicted)	95.00 (82.00; 107.00)	95.00 (84.00; 105.00)	0.17
FVC_ex_ (% of predicted)	97.00 (89.00; 107.00)	99.00 (89.00; 108.00)	0.40
PEF (% predicted)	71.00 (67.00; 92.00)	81.00 (65.00; 97.00)	0.45
MEF_75_ (% of predicted)	73.00 (65.00; 93.00)	74.00 (67.00; 102.00)	0.24
MEF_50_ (% of predicted)	80.00 (62.00; 91.00)	77.00 (54,00; 92,00)	0.27
MEF_25_ (% of predicted)	122.00 (94.00; 175.00)	121.00 (84.00; 147.00)	0.14

FEV_1_/FVC_ex_—forced expiratory volume during the first second of expiration to forced vital capacity; FEV_1_—forced expiratory volume during the first second of expiration; FVC_ex_—expiratory forced vital capacity; IQR—interquartile range; MEF_75_—maximal expiratory flow at 75% of FVC; MEF_50_—maximal expiratory flow at 50% of FVC; MEF_25_—maximal expiratory flow at 25% of FVC; PEF—peak expiratory flow. * *p*-value was assessed using the Wilcoxon signed-rank test. Data are expressed as medians (quartile 1; quartile 3).

## Data Availability

No new data were created or analyzed in this study. Data sharing is not applicable to this article.

## Supplementary Materials

The following supporting information can be downloaded at https://www.mdpi.com/article/10.3390/jcm14155431/s1, Table S1: Δ of assessed spirometry parameters before and 30 days after PVI in relation to BMI value; Table S2: Δ of assessed spirometry parameters before and 30 days after PVI in relation to the presence of CAD; Table S3: Δ of assessed spirometry parameters before and 30 days after PVI in relation to the presence of HF; Table S4 Δ of assessed spirometry parameters after PVI according to EF value; Table S5: Δ of assessed spirometry parameters after PVI in relation to the presence of T2DM; Table S6: Δ of assessed spirometry parameters after PVI in relation to the presence of DLP; Table S7: Δ of assessed spirometry parameters after PVI in relation to the presence of HA; Table S8: Δ of assessed spirometry parameters after PVI according to EHRA score; Table S9: Δ of assessed spirometry parameters PVI according to AF type.

## Abbreviations

The following abbreviations are used in this manuscript:

AF-PE	Persistent atrial fibrillation
AF	Atrial fibrillation
BMI	Body mass index
CAD	Coronary artery disease
CBA	Cryoballoon ablation
COPD	Chronic obstructive pulmonary disease
EF	Ejection fraction
FEV_1_	Forced expiratory volume during the first second of expiration
FEV_1_/FVC_ex_	Forced expiratory volume during the first second of expiration to forced vital capacity
FVC_ex_	Expiratory forced vital capacity
HF	Heart failure
MEF_25_	Maximal expiratory flow at 25% of FVC
MEF_50_	Maximal expiratory flow at 50% of FVC
MEF_75_	Maximal expiratory flow at 75% of FVC
PAF	Paroxysmal atrial fibrillation
PEF	Peak expiratory flow
PVI	Pulmonary vein isolation
TEE	Echocardiography
