# Supplementary material for: The Impact of Comorbidities on Pulmonary Function Measured by Spirometry in Patients After Percutaneous Cryoballoon Pulmonary Vein Isolation Due to Atrial Fibrillation
Source: J Clin Med. 2025 Aug 1;14(15):5431. doi: 10.3390/jcm14155431 (PMC12347115; doi:10.3390/jcm14155431)
Supplement: Supplementary file 1 [file jcm-14-05431-s001.zip › jcm-3762630-supplementary.pdf]

The impact of comorbidities on pulmonary function measured by spirometry in patients after percutaneous cryoballoon pulmonary vein isolation due to atrial fibrillation.

Table S1: Δ of assessed spirometry parameters before and 30 days after PVI in relation to the BMI value.

Spirometry parameter	BMI <30kg/m² (N		BMI ≥30kg/m²		*p**
	N	Median (Q1; Q3)	N	Median (Q1; Q3)	
Δ FEV_1/FVC_{ex} (%)	15	0.55 (-2.13; 1.08)	12	-0.25 (-2.38; 2.18)	0.90
Δ *FEV_1 (l)	15	-0.04 (-0.16; 0.22)	12	-0.11 (-0.28; 0.06)	0.21
Δ FVC_{ex} (l)	15	-0.12 (-0.24; 0.10)	12	-0.01 (-0.25; 0.12)	0.94
Δ PEF (l/s)	15	-0.04 (-0.34; 0.57)	12	-0.30 (-1.18; 0.76)	0.51
Δ MEF_{75} (l/s)	15	0.30 (0.10; 0.51)	12	-0.21 (-0.78; 0.30)	0.02
Δ MEF_{50} (l/s)	15	-0.13 (-0.61; 0.49)	12	-0.34 (-0.82; 0.18)	0.44
Δ MEF_{25} (l/s)	15	-0.07 (-0.21; 0.20)	12	-0.16 (-0.27; 0.05)	0.26
Δ FEV_1/FVC_{ex} (% of predicted)	15	0.00 (-3.00; 2.00)	12	-1.00 (-3.50; 2.50)	0.65
Δ FEV_1 (% of predicted)	15	0.00 (-5.00; 5.00)	12	-5.00 (-6.50; 1.00)	0.16
Δ FVC_{ex} (% of predicted)	15	-2.00 (-5.00; 6.00)	12	-2.00 (-7.00; 3.00)	0.52
Δ PEF (% predicted)	15	5.00 (-5.00; 11.00)	12	-3.50 (-15.00; 10.00)	0.29
Δ MEF_{75} (% of predicted)	15	5.00 (2.00; 9.00)	12	-3.50 (-10.50; 4.00)	0.02
Δ MEF_{50} (% of predicted)	15	-3.00 (-15.00; 15:00)	12	-7.50 (-17.00; 4.50)	0.52
Δ MEF_{25} (% of predicted)	15	-8.00 (-31.00; 35.00)	12	-18.50 (-30.00; 6.50)	0.44

BMI – body mass index, FEV1/FVC_{ex} – forced expiratory volume during the first second of expiration to forced vital capacity, FEV1 – forced expiratory volume during the first second of expiration, FVC_{ex} – expiratory forced vital capacity, MEF_{75} – maximal expiratory flow at 75% of FVC, MEF_{50} – maximal expiratory flow at 50% of FVC, MEF_{25} – maximal expiratory flow at 25% of FVC, PEF – peak expiratory flow,
* *p*-value assessed using Mann-Whitney U test.
The data are expressed as median (Quartile 1; Quartile 3).

Table S2: Δ of assessed spirometry parameters before and 30 days after PVI in relation to the presence of CAD.

Spirometry parameter	No-CAD		CAD		*p**
	N	Median (Q1; Q3)	N	Median (Q1; Q3)	
Δ FEV_1/FVC_{ex} (%)	21	0.12 (-1.77; 1.60)	6	0.09 (-2.82; 2.16)	0.88
Δ FEV_1 (l)	21	-0.06 (-0.23; 0.10)	6	-0.07 (-0.22; 0.09)	0.74
Δ FVC_{ex} (l)	21	-0.09 (-0.22; 0.10)	6	-0.11 (-0.37; 0.13)	0.54
Δ PEF (l/s)	21	-0.04 (-0.31; 0.73)	6	-0.38 (-0.88; 0.74)	0.30
Δ MEF_{75} (l/s)	21	0.30 (-0.30; 0.60)	6	-0.17 (-0.38; 0.01)	0.04
Δ MEF_{50} (l/s)	21	-0.22 (-0.79; 0.26)	6	-0.04 (-0.38; 0.19)	0.57
Δ MEF_{25} (l/s)	21	-0.13 (-0.21; 0.60)	6	-0.06 (-0.20; 0.16)	0.72
Δ FEV_1/FVC_{ex} (% of predicted)	21	-1.00 (-3.00; 2.00)	6	-0.50 (-4.00; 3.00)	0.86
Δ FEV_1 (% of predicted)	21	0.00 (-6.00; 3.00)	6	-4.50 (-6.00; 0.00)	0.39
Δ FVC_{ex} (% of predicted)	21	-1.00 (-4.00; 4.00)	6	-6.50 (-9.00; 4.00)	0.15
Δ PEF (% predicted)	21	5.00 (-4.00; 11.00)	6	-5.00 (-11.00; 5.00)	0.21
Δ MEF_{75} (% of predicted)	21	5.00 (-1.00; 9.00)	6	-3.50 (-08.00; -2.00)	0.03
Δ MEF_{50} (% of predicted)	21	-6.00 (-24.00; 7:00)	6	-2.50 (-11.00; 7.00)	0.83
Δ MEF_{25} (% of predicted)	21	-16.00 (-24.00; 7.00)	6	-9.50 (-33.00; 12.00)	0.95

CAD – coronary artery disease, FEV1/FVC_{ex} – forced expiratory volume during the first second of expiration to forced vital capacity, FEV1 – forced expiratory volume during the first second of expiration, FVC_{ex} – expiratory forced vital capacity, MEF_{75} – maximal expiratory flow at 75% of FVC, MEF_{50} - maximal expiratory flow at 50% of FVC, MEF_{25} – maximal expiratory flow at 25% of FVC, PEF – peak expiratory flow.
* *p*-value assessed using Mann-Whitney U test.
The data are expressed as median (Quartile 1; Quartile 3).

Table S3: Δ of assessed spirometry parameters before and 30 days after PVI in relation to the presence of HF.

Spirometry parameter	No-HF		HF		p*
	N	Median, (Q1; Q3)	N	Median, (Q1; Q3)	
Δ FEV_1/FVC_{ex} (%)	22	0.45 (-2.13; 0.85)	5	2.20 (2.16; 9.47)	0.05
Δ FEV_1 (l)	22	-0.06 (-0.23; 0.04)	5	0.09 (-0.22; 0.13)	0.61
Δ FVC_{ex} (l)	22	-0.10 (-0.23; 0.12)	5	0.01 (-0.37; 0.10)	0.77
Δ PEF (l/s)	22	-0.14 (-0.40; 0.79)	5	0.37 (-0.37; 0.54)	0.77
Δ MEF_{75} (l/s)	22	0.18 (-0.15; 0.60)	5	0.01 (-0.20; 0.42)	0.68
Δ MEF_{50} (l/s)	22	-0.30 (-0.79; 0.19)	5	0.17 (0.04; 0.49)	0.14
Δ MEF_{25} (l/s)	22	-0.13 (-0.21; 0.06)	5	0.04 (-0.17; 0.16)	0.49
Δ FEV_1/FVC_{ex} (% of predicted)	22	-1.00 (-3.00; 1.00)	5	3.00 (3.00; 12.00)	0.03
Δ FEV_1 (% of predicted)	22	-1.50 (-6.00; 2.00)	5	-8.00 (-9.00; 4.00)	0.85
/Δ FVC_{ex} (% of predicted)	22	-1.50 (-5.00; 4.00)	5	5.00 (-5.00; 6.00)	0.28
Δ PEF (% predicted)	22	-1.00 (-5.00; 12.00)	5	-3.00 (-4.00; 6.00)	0.57
Δ MEF_{75} (% of predicted)	22	3.00 (-3.00; 9.00)	5	-3.50 (-10.50; 4.00)	0.57
Δ MEF_{50} (% of predicted)	22	-7.00 (-18.00; 7:00)	5	1.00 (-7.00; 15.00)	0.13
Δ MEF_{25} (% of predicted)	22	-18.50 (-18.00; 7.00)	5	5:00 (-16.00; 8.00)	0.55

FEV_1/FVC_{ex} – forced expiratory volume during the first second of expiration to forced vital capacity, FEV_1 – forced expiratory volume during the first second of expiration, FVC_{ex} – expiratory forced vital capacity, HF – heart failure, MEF_{75} – maximal expiratory flow at 75% of FVC, MEF_{50} – maximal expiratory flow at 50% of FVC, MEF_{25} – maximal expiratory flow at 25% of FVC, PEF – peak expiratory flow

* p-value assessed using Mann-Whitney U test.

The data are expressed as median (Quartile 1; Quartile 3).

Table S4: Δ of assessed spirometry parameters after PVI according to the EF value.

Spirometry parameter	EF <50%		EF≥50%		p*
	N	Median (Q1; Q3)	N	Median (Q1; Q3)	
Δ FEV_1/FVC_{ex} (%)	6	2.80 (0.91; -9.82)	21	-0.62 (-2.48; 0.79)	0.02*
Δ FEV_1 (l)	6	0.65 (-0.30; 0.18)	21	-0.07 (-0.24; 0.05)	0.71
Δ FVC_{ex} (l)	6	0.06 (-0.54; 0.16)	21	-0.12 (-0.24; -0.11)	0.50
Δ PEF (l/s)	6	0.46 (-1.00; 1.30)	21	-0.17 (-0.64; 0.76)	0.76
Δ MEF_{75} (l/s)	6	0.22 (-0.34; 0.92)	21	0.17 (-0.19; 0.51)	0.72
Δ MEF_{50} (l/s)	6	0.11 (-0.35; 0.53)	21	-0.26 (-0.80; 0.23)	0.23
Δ MEF_{25} (l/s)	6	-0.07 (-0.02; 0.17)	21	-0.13 (-0.23; 0.13)	0.53
Δ FEV_1/FVC_{ex} (% of predicted)	6	3.00 (-1.50; 12.50)	21	-1.00 (-3.50; 1.00)	0.05
Δ FEV_1 (% of predicted)	6	-1.50 (-8.00; 6.00)	21	0.00 (-5.50; 2.50)	0.94
Δ FVC_{ex} (% of predicted)	6	-3.00 (-12.25; 4.50)	21	-2.00 (-5.00; 4.00)	0.47
Δ PEF (% predicted)	6	5.50 (-12.25; 13.00)	21	-2.00 (-7.00; 11.50)	0.94
Δ MEF_{75} (% of predicted)	6	1.50 (-5.50; 14.00)	21	2.00 (-4.00; 8.50)	0.82
Δ MEF_{50} (% of predicted)	6	1.00 (-7.25; 15.25)	21	-6.00 (-18.00; 8.00)	0.22
Δ MEF_{25} (% of predicted)	6	-3.00 (-18.75; 19.00)	21	-19.00 (-35.00; 14.00)	0.35

EF – ejection fraction, FEV_1/FVC_{ex} – forced expiratory volume during the first second of expiration to forced vital capacity, FEV_1 – forced expiratory volume during the first second of expiration, FVC_{ex} – expiratory forced vital capacity, MEF_{75} – maximal expiratory flow at 75% of FVC, MEF_{50} – maximal expiratory flow at 50% of FVC, MEF_{25} – maximal expiratory flow at 25% of FVC, PEF – peak expiratory flow.

* p-value assessed using Mann-Whitney U test.

The data are expressed as median (Quartile 1; Quartile 3).

Table S5: Δ of assessed spirometry parameters after PVI in relation to the presence of T2DM.

Spirometry parameter	No-T2DM		T2DM		p*
	N	Median (Q1; Q3)	N	Median (Q1; Q3)	
Δ FEV_1/FVC_{ex} (%)	16	-0.08 (-2.47; 0.96)	11	0.72 (-1.77; 7.53)	0.36
Δ FEV_1 (l)	16	-0.10 (-0.27; 0.03)	11	0.01 (-0.16; 0.13)	0.26
Δ FVC_{ex} (l)	16	-0.12 (-0.23; 0.08)	11	0.01 (-0.27; 0.13)	0.86
Δ PEF (l/s)	16	-0.20 (-0.37; 0.46)	11	0.54 (-0.87; 0.74)	0.74
Δ MEF_{75} (l/s)	16	0.13 (-0.11; 0.50)	11	0.20 (-0.38; 0.51)	0.94
Δ MEF_{50} (l/s)	16	-0.24 (-0.68; 0.16)	11	0.04 (-0.79; 0.49)	0.67
Δ MEF_{25} (l/s)	16	-0.13 (-0.29; 0.13)	11	-0.04 (-0.18; 0.16)	0.40
Δ FEV_1/FVC_{ex} (% of predicted)	16	-1.00 (-3.50; 1.00)	11	1.00 (-3.00; 9.00)	0.24
Δ FEV_1 (% of predicted)	16	-3.50 (-7.00; 2.00)	11	0.00 (-5.00; 3.00)	0.47
Δ FVC_{ex} (% of predicted)	16	-2.50 (-4.50; 4.00)	11	-1.00 (-8.00; 9.00)	0.65
Δ PEF (% predicted)	16	-2.50 (-5.00; 11.50)	11	6.00 (-9.00; 7.00)	0.91
Δ MEF_{75} (% of predicted)	16	3.00 (-2.50; 8.50)	11	2.00 (-8.00; 9.00)	0.71
Δ MEF_{50} (% of predicted)	16	-6.00 (-15.50; 5:50)	11	1.00 (-18.00; 15.00)	0.78
Δ MEF_{25} (% of predicted)	16	-18.50 (-37.00; 18.00)	11	-4.00 (-27.00; 12.00)	0.41

FEV_1/FVC_{ex} – forced expiratory volume during the first second of expiration to forced vital capacity, FEV_1 – forced expiratory volume during the first second of expiration, FVC_{ex} – expiratory forced vital capacity, MEF_{75} – maximal expiratory flow at 75% of FVC, MEF_{50} – maximal expiratory flow at 50% of FVC, MEF_{25} – maximal expiratory flow at 25% of FVC, PEF – peak expiratory flow, T2DM – type 2 diabetes mellitus.
* *p*-value assessed using Mann-Whitney U test.
The data are expressed as median (Quartile 1; Quartile 3).

Table S6: Δ of assessed spirometry parameters after PVI in relation to the presence of DLP.

Spirometry parameter	No-DLP		DLP		p*
	N	Median (Q1; Q3)	N	Median IQR (Q1; Q3)	
Δ FEV_1/FVC_{ex} (%)	9	-0.63 (-3.00; 0.72)	18	0.33 (-1.50; 3.40)	0.14
Δ FEV_1 (l)	9	-0.08 (-0.30; -0.06)	18	0.00 (-0.22; 0.09)	0.39
Δ FVC_{ex} (l)	9	-0.04 (-0.13; 0.05)	18	-0.13 (-0.28; 0.12)	0.36
Δ PEF (l/s)	9	-0.17 (-0.34; -0.04)	18	0.23 (-0.40; 0.79)	0.55
Δ MEF_{75} (l/s)	9	-0.10 (-0.23; 0.41)	18	0.24 (-0.15; 0.51)	0.55
Δ MEF_{50} (l/s)	9	-0.61 (-0.90; 0.06)	18	-0.04 (-0.38; 0.26)	0.15
Δ MEF_{25} (l/s)	9	-0.14 (-0.49; 0.06)	18	-0.12 (-0.20; 0.16)	0.45
Δ FEV_1/FVC_{ex} (% of predicted)	9	-1.00 (-4.00; 1.00)	18	-0.50 (-2.00; 3.00)	0.25
Δ FEV_1 (% of predicted)	9	-3.00 (-7.00; 0.00)	18	0.00 (-6.00; 3.00)	0.58
Δ FVC_{ex} (% of predicted)	9	-1.00 (-4.00; 6.00)	18	-3.50 (-7.00; 4.00)	0.18
Δ PEF (% predicted)	9	-2.00 (-5.00; 7.00)	18	5.50 (-5.00; 11.00)	0.95
Δ MEF_{75} (% of predicted)	9	2.00 (-5.00; 8.00)	18	3.00 (-3.00; 9.00)	0.58
Δ MEF_{50} (% of predicted)	9	-15.00 (-18.00; 1:00)	18	-1.00 (-8.00; 9.00)	0.15
Δ MEF_{25} (% of predicted)	9	-20.00 (-57.00; 7.00)	18	-13.50 (-24.00; 12.00)	0.30

DLP – dyslipidemia, FEV_1/FVC_{ex} – forced expiratory volume during the first second of expiration to forced vital capacity, FEV_1 – forced expiratory volume during the first second of expiration, FVCex – expiratory forced vital capacity, MEF_{75} – maximal expiratory flow at 75% of FVC, MEF_{50} – maximal expiratory flow at 50% of FVC, MEF_{25} – maximal expiratory flow at 25% of FVC, PEF – peak expiratory flow.
* *p*-value assessed using Mann-Whitney U test.
The data are expressed as median (Quartile 1; Quartile 3).

Table S7: Δ of assessed spirometry parameters after PVI in relation to the presence of HA.

Spirometry parameter	No-HA		HA		p*
	N	Median, (Q1; Q3)	N	Median (Q1; Q3)	
Δ FEV_1/FVC_{ex} (%)	6	0.77 (-2.13; 4.89)	21	-0.28 (-1.77; 1.60)	0.46
Δ FEV_1 (l)	6	-0.02 (-0.07; 0.13)	21	-0.08 (-0.23; 0.09)	0.57
Δ FVC_{ex} (l)	6	-0.12 (-0.17; 0.28)	21	-0.04 (-0.24; 0.10)	0.83
Δ PEF (l/s)	6	-0.17 (-0.34; 0.09)	21	-0.04 (-0.40; 0.79)	0.43
Δ MEF_{75} (l/s)	6	0.20 (0.10; 0.41)	21	0.17 (-0.20; 0.60)	0.97
Δ MEF_{50} (l/s)	6	0.05 (-0.22; 0.26)	21	-0.34 (-0.79; 0.19)	0.20
Δ MEF_{25} (l/s)	6	-0.03 (-0.17; 0.06)	21	-0.14 (-0.21; 0.16)	0.74
Δ FEV_1/FVC_{ex} (% of predicted)	6	1.50 (-3.00; 6.00)	21	-1.00 (-3.00; 1.00)	0.26
Δ FEV_1 (% of predicted)	6	1.00 (-3.00; 3.00)	21	-4.00 (-6.00; 2.00)	0.38
Δ FVC_{ex} (% of predicted)	6	-3.00 (-4.00; 6.00)	21	5.00 (-5.00; 12.00)	0.90
Δ PEF (% predicted)	6	-2.50 (-5.00; 5.00)	21	2.00 (-4.00; 9.00)	0.30
Δ MEF_{75} (% of predicted)	6	4.00 (2.00; 8.00)	21	-8.00 (-18.00; 7.00)	0.88
Δ MEF_{50} (% of predicted)	6	1.00 (-16.00; 9:00)	21	-18.00 (-31.00; 12.00)	0.18
Δ MEF_{25} (% of predicted)	6	-5.50 (-24.00; 7.00)	21	-13.50 (-24.00; 12.00)	0.81

FEV_1/FVC_{ex} – forced expiratory volume during the first second of expiration to forced vital capacity, FEV_1 – forced expiratory volume during the first second of expiration, FVC_{ex} – expiratory forced vital capacity, HA – hypertension arterialis, MEF_{75} – maximal expiratory flow at 75% of FVC, MEF_{50} – maximal expiratory flow at 50% of FVC, MEF_{25} – maximal expiratory flow at 25% of FVC, PEF – peak expiratory flow,
* p-value assessed using Mann-Whitney U test.
The data are expressed as median (Quartile 1; Quartile 3).

Table S8: Δ of assessed spirometry parameters after PVI according to EHRA score.

Spirometry parameter	EHRA 1-2		EHRA 3-4		p*
	N	Median (Q1; Q3)	N	Median (Q1; Q3)	
Δ FEV_1/FVC_{ex} (%)	11	-0.28 (-3.68; 1.08)	16	0.41 (-1.20; 2.80)	0.15
Δ FEV_1 (l)	11	-0.07 (-0.30; 0.02)	16	-0.02 (-0.19; 16.00)	0.37
Δ FVC_{ex} (l)	11	-0.09 (-0.19; 0.13)	16	-0.08 (-0.27; 0.11)	0.67
Δ PEF (l/s)	11	-0.17 (-1.17; 0.74)	16	0.02 (-0.35; 0.76)	0.53
Δ MEF_{75} (l/s)	11	0.10 (-0.23; 0.42)	16	0.18 (-0.17; 0.55)	0.67
Δ MEF_{50} (l/s)	11	-0.22 (-0.61; 0.17)	16	-0.12 (-0.77; 0.50)	0.74
Δ MEF_{25} (l/s)	11	-0.14 (-0.21; 0.16)	16	-0.12 (-0.20; 0.13)	0.86
Δ FEV_1/FVC_{ex} (% of predicted)	11	-1.00 (-4.00; 1.00)	16	-0.05 (-0.20; 2.50)	0.21
Δ FEV_1 (% of predicted)	11	-4.00 (-7.00; 0.00)	16	0.00 (-5.50; 4.00)	0.20
Δ FVC_{ex} (% of predicted)	11	-4.00 (-5.00; 6.00)	16	-1.50 (-5.50; 3.00)	0.88
Δ PEF (% predicted)	11	-2.00 (-13.00; 11.00)	16	2.50 (-5.00; 10.00)	0.63
Δ MEF_{75} (% of predicted)	11	2.00 (-5.00; 8.00)	16	3.00 (-2.50; 9.50)	0.38
Δ MEF_{50} (% of predicted)	11	-6.00 (-15.00; 1:00)	16	-3.00 (-17.00; 13.50)	0.80
Δ MEF_{25} (% of predicted)	11	-16.00 (-57.00; 8.00)	16	-14.50 (-25.50; 14.00)	0.53

EHRA – European Hearth Rhythm Association , FEV_1/FVC_{ex} – forced expiratory volume during the first second of expiration to forced vital capacity, FEV_1 – forced expiratory volume during the first second of expiration, FVC_{ex} – expiratory forced vital capacity, MEF_{75} – maximal expiratory flow at 75% of FVC, MEF_{50} – maximal expiratory flow at 50% of FVC, MEF_{25} – maximal expiratory flow at 25% of FVC, PEF – peak expiratory flow,
* p-value assessed using Mann-Whitney U test.
The data are expressed as median (Quartile 1; Quartile 3).

Table S9: Δ of assessed spirometry parameters PVI according to AF type.

**Spirometry parameter**	**PAF**		**Afib Pe**		***p****
	**N**	**Median (Q1; Q3)**	**N**	**Median (Q1:Q3)**	
Δ FEV_1/FVC_{ex} (%)	18	-0.45 (-2.13; 0.85)	9	1.60 (-1.77; 2.20)	0.55
Δ FEV_1 (l)	18	-0.06 (-0.25; 0.09)	9	-0.01 (-0.15; 0.09)	0.60
Δ FVC_{ex} (l)	18	-0.15 (-0.27; 0.09)	9	0.04 (-0.04; 0.12)	0.29
Δ PEF (l/s)	18	-0.07 (-0.40; 0.79)	9	-0.29 (-0.37; 0.73)	0.73
Δ MEF_{75} (l/s)	18	0.24 (-0.03; 0.42)	9	-0.08 (-0.81; 0.51)	0.22
Δ MEF_{50} (l/s)	18	-0.28 (-0.76; 0.06)	9	-0.17 (-0.47; 0.49)	0.34
Δ MEF_{25} (l/s)	18	-0.09 (-0.21; 0.06)	9	-0.14 (-0.20; 0.16)	0.95
Δ FEV_1/FVC_{ex} (% of predicted)	18	-1.00 (-3.00; 1.00)	9	0.00 (-3.00; 3.00)	0.79
Δ FEV_1 (% of predicted)	18	-1.50 (-7.00; 3.00)	9	-3.00 (-6.00; 2.00)	0.95
Δ FVC_{ex} (% of predicted)	18	-3.50 (-5.00; 4.00)	9	0.00 (-6.00; 4.00)	0.87
Δ PEF (% predicted)	18	2.50 (-5.00; 12.00)	9	-3.00 (-5.00; 7.00)	0.51
Δ MEF_{75} (% of predicted)	18	4.00 (-2.00; 8.00)	9	-3.00 (-11.00; 9.00)	0.24
Δ MEF_{50} (% of predicted)	18	-6.50 (-16.00; 1:00)	9	2.00 (-8.00; 12.00)	0.35
Δ MEF_{25} (% of predicted)	18	-12.00 (-31.00; 1.00)	9	-19.00 (-27.00; 8.00)	0.0.93

A-Fib PE – persistent atrial fibrillation, FEV_1/FVC_{ex} – forced expiratory volume during the first second of expiration to forced vital capacity, FEV_1 – forced expiratory volume during the first second of expiration, FVC_{ex} – expiratory forced vital capacity, MEF_{75} – maximal expiratory flow at 75% of FVC, MEF_{50} – maximal expiratory flow at 50% of FVC, MEF_{25} – maximal expiratory flow at 25% of FVC, PAF – paroxysmal atrial fibrillation, PEF – peak expiratory flow.

* *p*-value assessed using Mann-Whitney U test.

The data are expressed as median and IQR (Quartile 1; Quartile 3).
